# Physical Diagnosis*Abstract of a lecture recently delivered at Columbia Veterinary College.

**Published:** 1883-07

**Authors:** E. Benjamin Ramsdell

**Affiliations:** Lecturer on Diseases of the Respiratory Apparatus, Columbia Veterinary College and School of Comparative Medicine


### Art. XXI.—PHYSICAL DIAGNOSIS.*

BY E. BENJAMIN RAMSDELL, M.D.,

*Lecturer on Diseases of the Respiratory Apparatus, Columbia Veterinary College and School of Comparative Medicine.*

At our last lecture we discussed under the heading, "Palpation," the phenomena of friction fremitus and pulse, both in its relation to health and to diseased conditions.

* Abstract of a lecture recently delivered at Columbia Veterinary College.

To-day we will continue the study of the second method employed in Physical Diagnosis, namely: Palpation, by considering the subject of temperature. The sense of touch was at one time the only method, and among some older practitioners is now, employed for the detection of an increase of the bodily temperature; but science is not satisfied with such a crude mode of examination and it is better to use the more sensitive thermometer. The animal body is so constituted as to be able to withstand exposure to considerable degrees of heat and cold, and yet be in itself never much above or below the mean standard of health.

The animals that pant beneath the scorching rays of a tropical sun have the same mean temperature as the faithful creatures that accompany hazardous man in his search for the north pole.

This mean temperature is maintained in part by the action of the skin and its coverings, and partly through the agency of the nervous system in the regulation of combustion, &c., &c.

In man the temperature of health is 98½° Far. The mean temperature of the horse in health is about 99° Farenheit.

A slight elevation of temperature is not of great importance in itself, but in the incubative stage of glanders, pleuro-pneumonia and other contagious or infectious diseases its recognition is of the highest importance. Here, in the above mentioned cases, the early appreciation of the presence of the disease plays an important part in preventing the development and propagation of such maladies, and depends much upon the use of a delicate thermometer.

When an outbreak of such diseases as the above occurs, it is very possible that out of a large herd of cattle or stud of horses but one or two may present any appreciable sign of the disease, all the rest remaining apparently healthy; but although they may not give any evidence of the disease, it does not follow that they are not tainted with the malady in its incubative stage. We do not pretend that the simple rise of temperature is diagnostic of any particular disease, but, under the circumstances, it would be sufficient to indicate the necessity of quarantining such animals immediately.

The temperature should be taken in the rectum in every case for the sake of accuracy in comparison and for the safety of the instrument. The thermometer should be left in situ about four minutes.

The temperature in the rectum is generally conceded to be about one degree above that of the mouth, and for this fact there must be some correction made when we compare these results with temperatures taken in the mouth. * * *

All inflammatory diseases, such as pneumonia, pleurisy, synovitis, laminitis, etc., are accompanied by a rise in temper, ature, depending in great measure for its height upon the severity of the attack and the extent of surface involved.

Another very important result of palpation is the detection of accumulations of fluid by means of that peculiar sensation called fluctuation.

In the study of abdominal disease we quite frequently see large accumulations of serum in the peritoneal sac. This is called hydroperitoneum or ascites, and may be the concomitant of many diseases of both near and distant viscera. What is this ascites? How is it produced? Passive congestion of the local vessels is the main cause which, whether due to renal-cardiac, hepatic or peritoneal diseases, is always mechanical in its action.

It is produced in renal disease by an interference with the circulation in the minute vessels, arterioles capillaries and smaller veins through the medium of the vasa motor nerves, whose centers have been irritated by the retained materies morbi; the product of tissue metamorphosis; urea.

In cardiac disease it occurs as the result of failure on the part of the right ventricle to carry the blood onward: and this failure allows the veins, notably those of the abdominal viscera, to become distended with the sluggish current.

In hepatic disease it is due to pressure upon the vessels which diminishes their calibre and so dams back the blood. This is a very prominent cause of ascites. By this passive congestion, which I repeat, is purely mechanical, the capillaries become over distended and the serum of the blood extends. This effused serum is of less specific gravity than the serum of the blood; but if the pressure within the capillaries be increased, the exudation becomes of a higher specific gravity and more and more like the liquor sanguinis. In some cases the cause is disease of the peritoneum. A chronic peritonitis due to malignant growths may induce a deposit of serous fluid in this peritoneal cavity.

In every case of ascites the intestines, more or less distended with gas, float high up in the abdomen and the lower zone is occupied only by serum. Placing the open palm of the hand on one side of the animal's abdomen, and then striking a few short, quick blows on the other side we will feel as though the blows were struck upon our palm. These taps are the blows of the wave which has been carried by the fluid from one side over to the hand on the other side.

This is a positive indication of the presence of fluid, and once felt will always be recognized whenever it exists.

In cases of localized inflammation, when you suspect there is a collection of pus, it is by this principle and this manouvre that its presence can be detected.

Do not dig your fingers down into the tissues first on one side of the swelling and then on the other, as I have seen many do; but hold one hand smoothly and firmly on one side of the tumefaction while you tap the surface gently on the other side with the fingers of the other hand. This is the only proper way to detect the presence of fluid collections by palpation.

After having examined our patient by inspection and palpation, having noted the number and quality of the respirations, the number and characteristics of the pulse, the temperature, &c., &c., we next bring into the field the third method of Physical Diagnosis, percussion.

This, one of the most important methods employed in Diagnosis, is the process by which we elicit sound by striking blows on the chest wall. We have two varieties of percussion: first, the immediate; and second, the mediate percussion. In the first variety the blow is made with the fingers, knuckles, or hammer directly upon the chest wall without any substance being interposed; therefore it is called the *immediate* variety. In the second, or *mediate* percussion we *interpose* between the chest wall and the fingers, knuckles, or hammer some intervening substance like the finger, or a plate of ivory, bone, hard rubber, maleable glass, etc., etc. This intermediate substance is called the pleximeter, while the object with which the sound is produced is called the percussion hammer.

In order to understand the results of percussion, or in other words be able to appreciate the percussion note, you must first somewhat clearly comprehend the elements of sound.

In general we may say that sounds differ in four ways. These peculiarities of sound are intensity, duration, quality and pitch. All these four characteristics are important in studying a percussion note; but the principle one is pitch.

Intensity means loudness, and this may be increased or diminished by increasing or diminishing the force of the percussion blow. Nevertheless in pulmonary percussion you will find that the intensity is also modified by the amount of air contained in the lung tissue, by the thickness of the soft parts covering the thoracic walls, and by the thickness of the coating of hair.

Duration of sound has reference to the length of time the sound takes to produce its effect upon our auditory apparatus.

One sound seems short and quick, another appears to take a long time in coming and fades gradually away. This of course is an extreme example. We find, as a matter of fact,: that other things being equal the pitch regulates the duration the higher the pitch the shorter the duration, and the lower, the pitch the correspondingly longer is the duration.

We define quality as that peculiarity of sound by which we appreciate the difference between a note on one musical instrument and the same note produced by a different instrument.

In sounds produced by percussion you can readily appreciate the difference between this sound produced by a blow on a wooden table, which we say has a wooden quality; this sound produced by a blow on a distended tympanitic abdomen, which sound we call tympanitic or drum-like in quality; and a third sound produced by percussion over normal lung tissue with properly distended air vesicles, which sound, we consider has a vesicular quality.

Pitch, the most important peculiarity of sound in percussion, is the position of the note on the musical scale; and we designate a note on the scale by saying that its pitch is high or low.

Some men's voices are pitched very low; women's voices are usually pitched much higher. A soprano voice has a range among the high pitched notes while a basso voice uses the notes which are low pitched.

By this imperfect description you will, I hope, obtain a clear idea of the difference between sounds as regards their pitch.

In studying percussion we take for our standard the sound obtained by percussing over healthy lung tissue, when all the conditions are favorable, such as a thin layer of hair, only a moderate thickness of muscular coating, etc., etc.

The percussion note thus obtained is called the normal pulmonary vesicular resonance, and cannot be described in a word-picture; but can only be studied by listening to the sound when produced under proper conditions and carefully considering its intensity, its duration, its quality and its pitch. After you have imprinted this sound thoroughly in your memory, you may with profit listen to abnormal percussion sounds, comparing them with the normal resonance.

We speak of one percussion sound as being more or less intense than the normal resonance. We call the sound longer or shorter than the normal as regards its duration. We speak of a hard, a soft, a metalic, or a wooden sound, as regards its quality, and as regards pitch we have higher or lower sounds than the normal pulmonary vesicular resonance. Of the various sounds obtained by percussion over the chest in health and disease seven have received special names.

The accompanying table gives these different sounds with their relative musical characteristics and the different conditions under which they are produced.

| Percussion Sound. | Pitch. | Duration. | Quality. | Intensity. | Conditions Producing the Sound. |
|---|---|---|---|---|---|
| Tympanitic resonance, | 1 | 7 | Drum-like | 5 | Pneumo-thorax. |
| Vesiculo-tympanitic resonance, | 2 | 6 | " | 4 | Emphysema. |
| Exaggerated pulmonary vesicular resonance, | 3 | 5 | Vesicular | 3 | Vicarious emphysema. |
| Normal pulmonary vesicular resonance, | 4 | 4 | " | 2 | Normal lung tissue. |
| Dullness, | 5 | 3 | Wooden | 1 | Consolidation of lung tissue. |
| Flatness, | 6 | 2 | Muscular | 3 | Absence of lung tissue. Tumor or fluid. |
| Amphoric resonance, | 7 | 1 | Metalic | 5 | Cavitie in lung tissue. |

Although you may notice many defects, yet I think this table may be of much practical value to you in the study of respiratory diseases.

The tympanitic resonance is a full, round, drum-like sound and can be obtained by percussing over an abdomen whose intestines are filled with gas. That is the typical sound. Its acoustic properties are, as compared with the normal pulmonary vesicular resonance, its pitch is much lower, its intensity is much increased, its duration is longer, and its quality is drum-like. All these facts and characteristics you will notice are shown in the table.

In thoracic percussion in order to obtain this sound the air must not be in small compartments like the air vesicles of a healthy lung, but must be present in large collections; therefore the only condition productive of this percussion sound would be pneumo-thorax. Pneumo-thorax is a diseased condition in which the pleura no longer has its two surfaces, visceral and parietal, in contact; but the whole sac is distended with air and the lung is collapsed. This disease will be discussed more fully when we come to consider the diseases proper of the lungs.

The vesiculo-tympanitic resonance is so called because it partakes of the properties, both of the tympanitic and of the normal vesicular resonance.

Its pitch is a little lower and its intensity is a little greater than those of the normal resonance, while its duration is shorter than, and its quality similar to, the tympanitic resonance. It is the sound obtained by percussion over a lung whose air vesicles are abnormally distended with air, and there is an increase in the volume of the lung. This condition, where one air space occupies the position formerly filled by several smaller air vesicles, is the result of emphysema; and the vesiculo-tympanitic resonance is the characteristic sound of this disease.

The exaggerated pulmonary vesicular resonance is a sound which differs from the normal solely in a slight increase in its intensity and duration, and a little lowering of the pitch: the quality still remains vesicular. This sound is heard when we percus over lung tissue which is doing double duty. In cases of pneumonia or plurisy with effusion where one lung is much obstructed and interferred with in its duty, we have the healthy lung doing so much increased work: aerating so much more than its usual compliment of blood, both by increasing the rapidity and number of its respiratory efforts, but also by increasing the extent of its aerating surface; that we obtain this exaggerated vesicular resonance.

The normal pulmonary vesicular resonance is obtained over healthy lung tissue which is doing its proper share of duty. We take this sound for our standard and the first thing we must all learn is this normal sound which is the same whether obtained by percussion on the thorax of a man, a horse, a dog, or a cat.

Dullness is that peculiarity of the percussion note in which, as compared with the normal sound, the pitch is higher, the intensity is less, the duration is shortened and the quality is hardened.

Dullness may be slight, considerable, or complete, according as more or less air enters that portion of the lung tissue under examination.

It always indicates a decrease in the amount of air in the part and is an important physical sign in the detection of Pnemonia, Tuberculosis, Œdema of the lungs, etc; where the air vesicles are filled with an exudation to the more or less complete exclusion of air.

Flatness indicates a total absence of air. so that there is no pulmonary resonance. It is the resonance we obtain when we percuss over the liver or over that thick mass of muscles on the foreshoulder. The musical peculiarities of this sound are, that the quality is hard, fleshy or muscular, as we have called it in our table; its pitch is high, its duration is short, and its intensity is great.

This sound is obtained over fluid either in the pleural, pericardial or peritoneal sac, or over tumors either solid or fluid which may invade the thoracic or abdominal cavity, and also in some cases of extensive inflammatory thickening and induration of the pleuræ.

